# The three-dimensional structure of *Drosophila melanogaster* (6–4) photolyase at room temperature

**DOI:** 10.1107/S2059798321005830

**Published:** 2021-07-29

**Authors:** Andrea Cellini, Weixiao Yuan Wahlgren, Léocadie Henry, Suraj Pandey, Swagatha Ghosh, Leticia Castillon, Elin Claesson, Heikki Takala, Joachim Kübel, Amke Nimmrich, Valentyna Kuznetsova, Eriko Nango, So Iwata, Shigeki Owada, Emina A. Stojković, Marius Schmidt, Janne A. Ihalainen, Sebastian Westenhoff

**Affiliations:** a Department of Chemistry and Molecular Biology, University of Gothenburg, Box 462, 405 30 Gothenburg, Sweden; b Physics Department, University of Wisconsin-Milwaukee, 3135 North Maryland Avenue, Milwaukee, WI 53211, USA; c Department of Biological and Environmental Science, Nanoscience Center, University of Jyvaskyla, 40014 Jyvaskyla, Finland; d Department of Anatomy, Faculty of Medicine, University of Helsinki, Box 63, 00014 Helsinki, Finland; e Department of Cell Biology, Graduate School of Medicine, Kyoto University, Yoshidakonoe-cho, Sakyo-ku, Kyoto 606-8501, Japan; f RIKEN SPring-8 Center, 1-1-1 Kouto, Sayo-cho, Sayo-gun, Hyogo 679-5148, Japan; g Japan Synchrotron Radiation Research Institute, 1-1-1 Kouto, Sayo-cho, Sayo-gun, Hyogo 679-5198, Japan; h Institute of Multidisciplinary Research for Advanced Materials, Tohoku University, 2-1-1 Katahira, Aoba-ku, Sendai 980-8577, Japan; i Department of Biology, Northeastern Illinois University, 5500 North St Louis Avenue, Chicago, IL 60625, USA

**Keywords:** photolyases, flavoproteins, FAD, serial crystallography, room-temperature structure, *Drosophila melanogaster*, (6–4) photolyase

## Abstract

(6–4) photolyases are flavoproteins that belong to the photolyase/cryptochrome family. Their function is to repair DNA lesions using visible light. Here, crystal structures of *Drosophila melanogaster* (6–4) photolyase [*Dm*(6–4)photolyase] at room and cryogenic temperatures are reported. The room-temperature structure was solved to 2.27 Å resolution and was obtained by serial femtosecond crystallography (SFX) using an X-ray free-electron laser. The crystallization and preparation conditions are also reported. The cryogenic structure was solved to 1.79 Å resolution using conventional X-ray crystallo­graphy. The structures agree with each other, indicating that the structural information obtained from crystallography at cryogenic temperature also applies at room temperature. Furthermore, UV–Vis absorption spectroscopy confirms that *Dm*(6–4)photolyase is photoactive in the crystals, giving a green light to time-resolved SFX studies on the protein, which can reveal the structural mechanism of the photoactivated protein in DNA repair.

## Abstract 2

A crystal structure of a photolyase at room temperature confirms the structural information obtained from cryogenic crystallography and paves the way for time-resolved studies of the photolyase at an X-ray free-electron laser.

## 1. Introduction   

Light plays an important role in plant and animal physiology. To name a few examples, light regulates development, de-etiolation and flowering in plants (Liu *et al.*, 2017 ▸; Putterill *et al.*, 2004 ▸), and vision and vitamin D activation in animals (Holick, 2000 ▸; Lythgoe, 1984 ▸). However, the ultraviolet content of sunlight can also damage DNA, causing mutagenic and cytotoxic effects (Lo *et al.*, 2005 ▸; LeClerc *et al.*, 1991 ▸). To compensate for this, archaea, prokaryotes and eukaryotes have photolyase proteins, which repair damaged DNA using the energy of visible light (Sancar & Rupert, 1978 ▸; Sancar *et al.*, 1987 ▸).

Photolyases are members of the cryptochrome/photolyase photoreceptor family (Kavakli *et al.*, 2017 ▸; Todo, 1999 ▸; Deisenhofer, 2000 ▸). While photolyases repair DNA, cryptochromes are circadian photoreceptors in plants that regulate growth and development (Emery *et al.*, 1998 ▸; Stanewsky *et al.*, 1998 ▸; Crane & Young, 2014 ▸). Cryptochromes also establish the circadian clock in insects and are thought to control the magnetic compass in birds (Ritz *et al.*, 2000 ▸; Dodson *et al.*, 2013 ▸; Hore & Mouritsen, 2016 ▸; Sheppard *et al.*, 2017 ▸). In mammals, cryptochromes are also involved in the circadian clock, but in a light-independent way (Griffin *et al.*, 1999 ▸). Despite their very different functions in organisms, photolyases and cryptochromes share a similar architecture and use a similar mode of photoexcitation.

**Figure 1:**
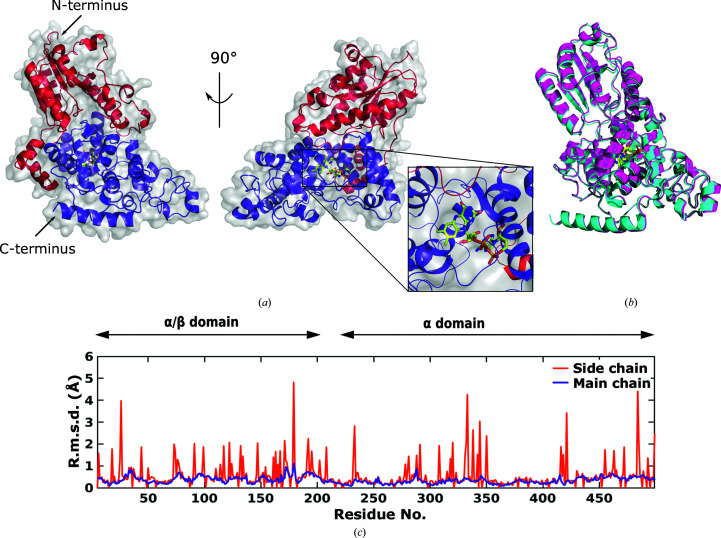
Overview of the 3D structure of *Dm*(6–4)PL at cryogenic and ambient temperatures. *(*a*) Dm*(6–4)PL structure solved at 1.79 Å resolution from data collected at cryo-temperature (PDB entry 7ayv). The α/β domain is depicted in red and the α domain is depicted in blue. The inset highlights the FAD in the α domain. (*b*) Superimposition of PDB entry 7ayv and the *Dm*(6–4)PL structure solved at 2.27 Å resolution from data collected at room temperature (PDB entry 7azt). The cryogenic structure is depicted in blue and the room-temperature structure is depicted in magenta. (*c*) R.m.s.d. of the main chain (blue) and the side chain (orange) in the room-temperature structure with the cryo-structure used as a reference.

Structurally, photolyases and cryptochromes have an N-terminal α/β domain and a C-terminal α-helical domain (Figs. 1 ▸ *a* and 1 ▸ *b*). Cryptochromes have an additional C-terminal tail (CTT) in the α-helical domain, which differentiates protein function between cryptochromes and photolyases (Chaves *et al.*, 2006 ▸). The α-helical C-terminal domain harbors a flavin adenine dinucleotide (FAD) chromophore (Fig. 1 ▸ *a*). The redox-active FAD takes part in light-induced reactions that lead to photoactivation.

The photoactivation mechanism is conserved between photolyases and cryptochromes. FAD absorbs blue light and initiates a cascade of electron-transfer reactions along a conserved tryptophan tetrad (triad in some members), yielding 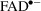 + 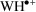 . This tetrad of tryptophans is highly conserved in the photolyase and cryptochrome family and is essential for spatially separating the radical pair (Dodson *et al.*, 2013 ▸; Yamamoto *et al.*, 2017 ▸; Aubert *et al.*, 2000 ▸; Popović *et al.*, 2002 ▸). In many members of the family, the terminal and solvent-exposed tryptophan releases a proton to the surrounding solvent and a more stable 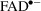 – 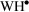 radical pair forms. In cryptochromes, the radical 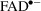 activates the protein and triggers downstream function, likely through transduction through the CTT (Berntsson *et al.*, 2019 ▸; Vaidya *et al.*, 2013 ▸). In photolyases, the neutral radical 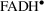 can be reduced a second time by blue light, preparing the fully reduced FADH^−^– 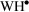 , primed for the repair of DNA (Henbest *et al.*, 2008 ▸).

The light-driven electron- and proton-transfer reactions of (6–4)photolyases and cryptochromes have been studied by time-resolved spectroscopy (Martin *et al.*, 2017 ▸; Li *et al.*, 2010 ▸; Lacombat *et al.*, 2019 ▸). These studies have revealed the sequence, the energetics and the kinetics of the reactions. Infrared spectroscopy points to complex structural rearrangements, including light-dependent unfolding in the α/β domain (Thöing *et al.*, 2015 ▸). Time-resolved solution scattering has provided structural insight into the release of the CTT in cryptochromes (Berntsson *et al.*, 2019 ▸), in agreement with static SAXS structures of cryptochromes (Vaidya *et al.*, 2013 ▸). The named techniques offer flexibility and time-resolution, and in some cases even structural insight (Takala *et al.*, 2014 ▸, 2016 ▸; Henry *et al.*, 2020 ▸; Ravishankar *et al.*, 2020 ▸; Berntsson *et al.*, 2017 ▸), but the structural specificity is usually limited. Time-resolved crystallography is able to reveal more specific information about how the protein responds to photoexcitation in photolyases and cryptochromes.

Here, we studied the *Drosophila melanogaster* (6–4) photolyase [*Dm*(6–4)PL] to investigate the common photoexcitation mechanism in (6–4) photolyases and cryptochromes. The photoreceptor protein uses near-UV and visible light (300–500 nm) as an energy source to monomerize (6–4)TT [or (6–4)CC] lesions in DNA (Kim & Sancar, 1993 ▸). The tryptophan tetrad consists of Trp407, Trp384, Trp330 and Trp381. The structure of *Dm*(6–4)PL in complex with DNA has been solved (PDB entries 2wb2, 3cvu and 3cvy) by crystallography at cryogenic temperatures (Maul *et al.*, 2008 ▸; Glas *et al.*, 2009 ▸). To the best of our knowledge, a crystal structure of a (6–4) photolyase at room temperature has not yet been reported. Static X-ray crystallographic structures of photolyases without and with damaged DNA suggest complex conformational rearrangements (Kiontke *et al.*, 2011 ▸; Mees *et al.*, 2004 ▸), but the mechanism remains poorly understood at the atomic level. FAD reduction and DNA repair have been reported after exposure to X-rays (Kort *et al.*, 2004 ▸). The DNA-repair activity of photolyase triggered by light decreases dramatically at temperatures below 200 K (Langenbacher *et al.*, 1997 ▸). Together, this makes it challenging to study the structural photoresponse of the protein and the process of DNA repair by cryo-crystallography.

Time-resolved serial femtosecond crystallography (SFX) at room temperature is a viable alternative. Here, a stream of microcrystals of the protein are supplied to the X-ray beam either in a jet, in a capillary or deposited on a fixed substrate plate. The method can be combined with short or continuous laser excitation (Tenboer *et al.*, 2014 ▸). When ultrashort X-ray pulses from free-electron lasers are used, it is possible to record diffraction before the high radiation dose changes the structure of the protein (Neutze *et al.*, 2000 ▸). For these reasons, SFX is ideal for studying photoactivation processes in cryptochromes/photolyases. Here, we establish protocols for the preparation of microcrystals of *Dm*(6–4)PL, demonstrate that the proteins in the crystals are light-active and solve a structure of the protein at room temperature. With this, we pave the way for time-resolved experiments on *Dm*(6–4)PL light activation in future SFX experiments.

## 2. Materials and methods   

### 2.1. *Dm*(6–4)PL expression and purification   

The *Dm*(6–4)PL plasmid was a gift from Dr Markus Müller (Ludwig Maximilian University of Munich). The protein was expressed and purified as reported previously (Maul *et al.*, 2008 ▸). Briefly, the plasmid was transformed in *Escherichia coli* Rosetta-gami cells (Merck) and the cells were grown at 37°C in Terrific Broth medium supplemented with 50 µg ml^−1^ carbenicellin and 17 µg ml^−1^ chloramphenicol. When the optical density at 600 nm reached 1.0, protein expression was induced by the addition of 200 ng ml^−1^ anhydrotetracycline (Sigma–Aldrich) at 18°C for 14–16 h. The cells were lysed and homogenized in a high-pressure homogenizer (Emulsiflex C3, Avestin). The cell lysate was treated with DNAseI for 1 h and loaded onto a StrepTrap HP column (GE Healthcare), followed by a HiTrap Heparin HP affinity column (GE Healthcare) and a Superdex 200 pg 16/600 column. Each step was performed under safe red-light conditions. The purification quality was assessed at each step by sodium dodecyl sulfate polyacrylamide gel electrophoresis (SDS–PAGE). The protein concentration was determined using the molar extinction coefficient of FAD at 445 nm (11.3 m*M* ^−1^ cm^−1^), whereas the protein function was evaluated by measuring its photoconversion ability after blue-light exposure.

### 2.2. *Dm*(6–4)PL macrocrystallization   

Initial crystallization conditions were identified using various commercially available crystallization screens, and the hit (100 m*M* bis-Tris pH 6.5, 200 m*M* lithium sulfate monohydrate, 25% PEG 3350) was further optimized using Additive Screen (Hampton Research). The addition of 4%(*v*/*v*) polypropylene glycol P400 to the crystallization condition improved the diffraction quality significantly. Under these conditions, crystals reached dimensions of 55 × 55 × 1000 µm within two days at 20°C. Prior to flash-cooling, the crystals were transferred to a solution of mother liquor and PEG 400 in a 1:1 ratio.

### 2.3. *Dm*(6–4)PL microcrystallization   

In order to reduce the crystal size to 10–50 µm for serial crystallography, we crystallized the protein at 4°C using seeds obtained from crushed macrocrystals. This increases the probability of multiple nucleation sites. Macrocrystals were crushed with beads from a Seed Bead kit (Hampton Research) and the crystal slurry was used as seeds for microcrystal growth in a batch setup. A 100 µl microcrystal batch was prepared by the addition of 10%(*v*/*v*) seeds to a mixture of 40%(*v*/*v*) protein (15 mg ml^−1^), 40%(*v*/*v*) precipitant and 10%(*v*/*v*) polypropylene glycol P400. The batch was left on a rocking table at 4°C for two days. The crystals were examined under a microscope and overall their size ranged between 20 and 40 µm. Prior to loading the sample into the sample injector (Shimazu *et al.*, 2019 ▸), the crystals were concentrated and subsequently dispersed 1:10 in highly viscous nuclear-grade grease, for example Super Lube Nuclear Grade (Synco Chemical Corp.). The sample was immediately used for data collection.

### 2.4. Data acquisition and analysis   

X-ray data from macrocrystals were collected at cryogenic temperature (100 K) on the BioMAX beamline at MAX IV Laboratory, Sweden. The wavelength of the X-rays was 0.9184 Å (13.5 keV). BioMAX has a flux of 2 × 10^13^ photons s^−1^. 15% X-ray beam transmission was used; the total exposure time was 40 s and the spot size of the X-rays was 50 × 50 µm. This corresponds to an average diffraction-weighted dose of 3.2 MGy, which we estimated using *RADDOSE*-3*D* (Zeldin *et al.*, 2013 ▸). The data were processed with the *XDS* package (Kabsch, 2010 ▸) and the structure was solved to 1.79 Å resolution using PDB entry 3cvy (Maul *et al.*, 2008 ▸) as a search model in *Phenix* (Liebschner *et al.*, 2019 ▸).

Room-temperature SFX data were collected from *Dm*(6–4)PL microcrystals on beamline BL3 at the SPring-8 Angstrom Compact free electron LAser (SACLA), Japan (Ishikawa *et al.*, 2012 ▸; Tono *et al.*, 2013 ▸; for additional information, see Claesson *et al.*, 2020 ▸). 200 µl of the microcrystal–grease mixture was transferred into an injector reservoir and the reservoir was installed in the diffraction chamber filled with helium (Tono *et al.*, 2015 ▸). The microcrystals were extruded through a 100 µm diameter nozzle at a flow rate of about 4.2 µl min^−1^ at ambient temperature. X-ray pulses of 7 keV were delivered to the microcrystal–grease mixture with a repetition rate of 30 Hz (Kameshima *et al.*, 2014 ▸). The collected frames were first sorted by *Cheetah* (Barty *et al.*, 2014 ▸; Nakane *et al.*, 2016 ▸) and were subsequently indexed and integrated in *CrystFEL* (version 0.6.3; White *et al.*, 2012 ▸). The indexing was performed using *Dirax* and *MOSFLM* (Battye *et al.*, 2011 ▸; Duisenberg, 1992 ▸). The data were merged and scaled using *partialator* in *CrystFEL* to produce an *hkl* file. A total of 209 242 diffraction images were collected at SACLA. The hit rate was about 5.74% and the indexing rate was 85.72%. The structure was solved to 2.27 Å resolution using the cryogenic structure as a search model in *Phenix*.

The models at cryo-temperature and ambient temperature were refined using *phenix.refine*. *SUPERPOSE* from the *CCP*4 suite was used to analyse the r.m.s.d. for structural comparison of these two models (Krissinel & Henrick, 2004 ▸). The statistics of data collection, processing, structure determination and refinement of both data sets are summarized in Table 1 ▸. All structure figures were generated using *PyMOL* (Schrödinger). The structures were deposited in the Protein Data Bank (PDB entries 7ayv and 7azt).

### 2.5. UV–visible absorbance spectroscopy   

Absorption spectra of *Dm*(6–4)PL in solution were recorded after the size-exclusion column purification step using a NanoDrop ND-1000 UV–Vis Spectrophotometer (Thermo Fisher). The UV–Vis spectra were recorded in the dark and after tens of seconds of exposure to LED light at 455 nm wavelength at approximately 116 mW cm^−2^.

UV–Vis spectra of the microcrystals were recorded using an AvaSpec-ULS2048CL-EVO (Avantes) spectrometer in combination with an AvaLight-XE (Avantes) light source. The microcrystals were rinsed with mother liquor and loaded into a quartz capillary with an inner diameter of 0.3 mm. Spectra were recorded in the dark and during illumination with LED light at 455 nm at 4.2 mW cm^−2^. All spectra were measured at room temperature and at wavelengths of 300–600 nm.

## 3. Results   

**Figure 2:**
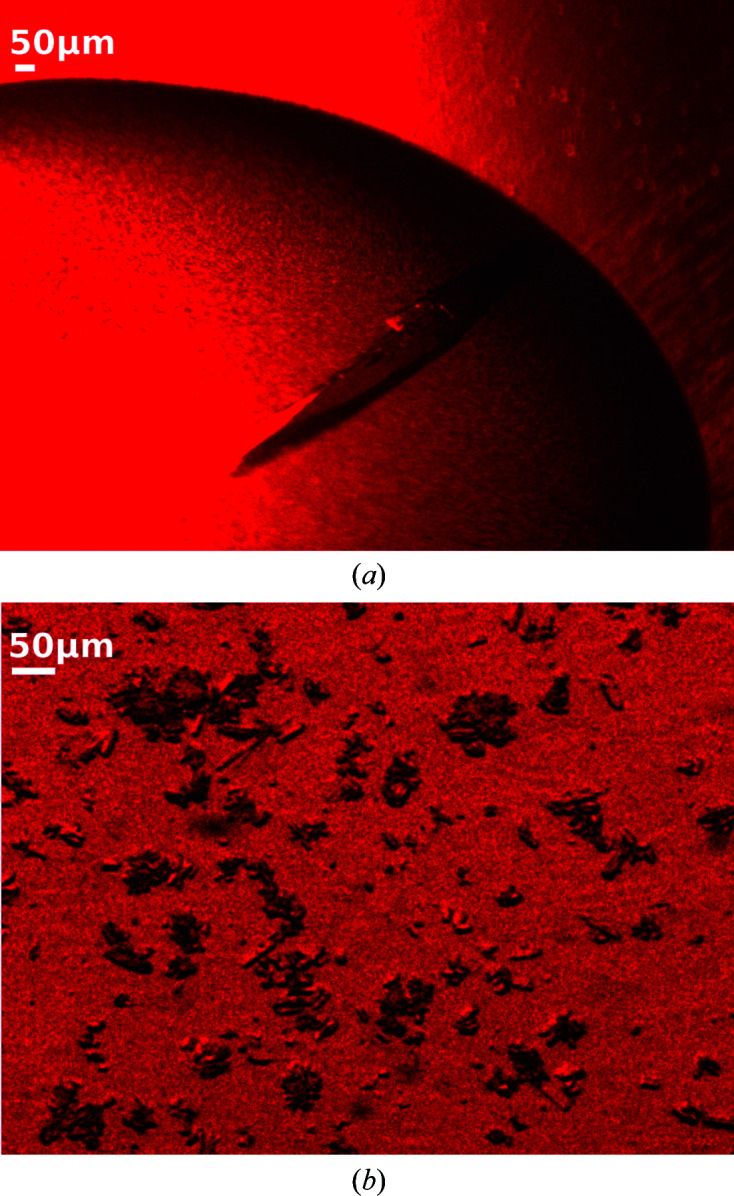
Crystals of *Dm*(6–4)photolyase. (*a*) A *Dm*(6–4)PL macrocrystal and (*b*) microcrystals.

We report new crystallization conditions for *Dm*(6–4)PL macrocrystals, which produced crystals with dimensions of 55 × 55 × 1000 µm within two days at 20°C (Fig. 2 ▸ *a*). We solved the structure to 1.79 Å resolution by single-crystal crystallo­graphy at cryogenic temperature. For SFX experiments, we developed a batch crystallization protocol in combination with a seeding method and successfully produced crystals with a homogeneous size distribution of 20–40 µm (Fig. 2 ▸ *b*). Using a viscous jet at SACLA, the grease–microcrystal mixture allows slow flow rates, minimizing sample consumption (Sugahara *et al.*, 2015 ▸). Using only 975 µg of protein, we recorded 209 242 diffraction images with a total acquisition time of about 2 h. From these, we solved the room-temperature structure of *Dm*(6–4)PL to 2.27 Å resolution (Table 1 ▸).

Both of our structures of *Dm*(6–4)PL reproduce the previously published structure (PDB entry 3cvy; Maul *et al.*, 2008 ▸). The structures display a globular shape with two main domains. The N-terminal domain (residues 1–203) has seven α-helices enclosing a core of five parallel β-strands and resembles a Rossmann fold (Hitomi *et al.*, 2009 ▸). The α-helical domain is located at the C-terminus and delimits an amphipathic pocket that harbors the FAD. An interconnecting region (residues 204–223) tethers the two domains.

Our structure from SFX at room temperature is highly similar to the cryogenic structure (Fig. 1 ▸ *b*). Root-mean-square deviation (r.m.s.d.) analysis (using *SUPERPOSE* from *CCP*4) confirms this. Indeed, the mean r.m.s.d. calculated on the main-chain residues is 0.35 Å, which indicates that the position of the main chains was unchanged. However, some of the positions of the side chains change at the higher temperature (Fig. 1 ▸ *c*). The residues with high r.m.s.d. values for side chains are located either on surface regions or loop regions. The unit-cell dimensions are slightly larger at room temperature than under cryogenic conditions by about 1 to 2 Å in each dimension (Table 1 ▸). This can be explained by thermal lattice expansion at ambient temperature. We conclude that the structure of *Dm*(6–4)PL is preserved at room temperature.

**Figure 3:**
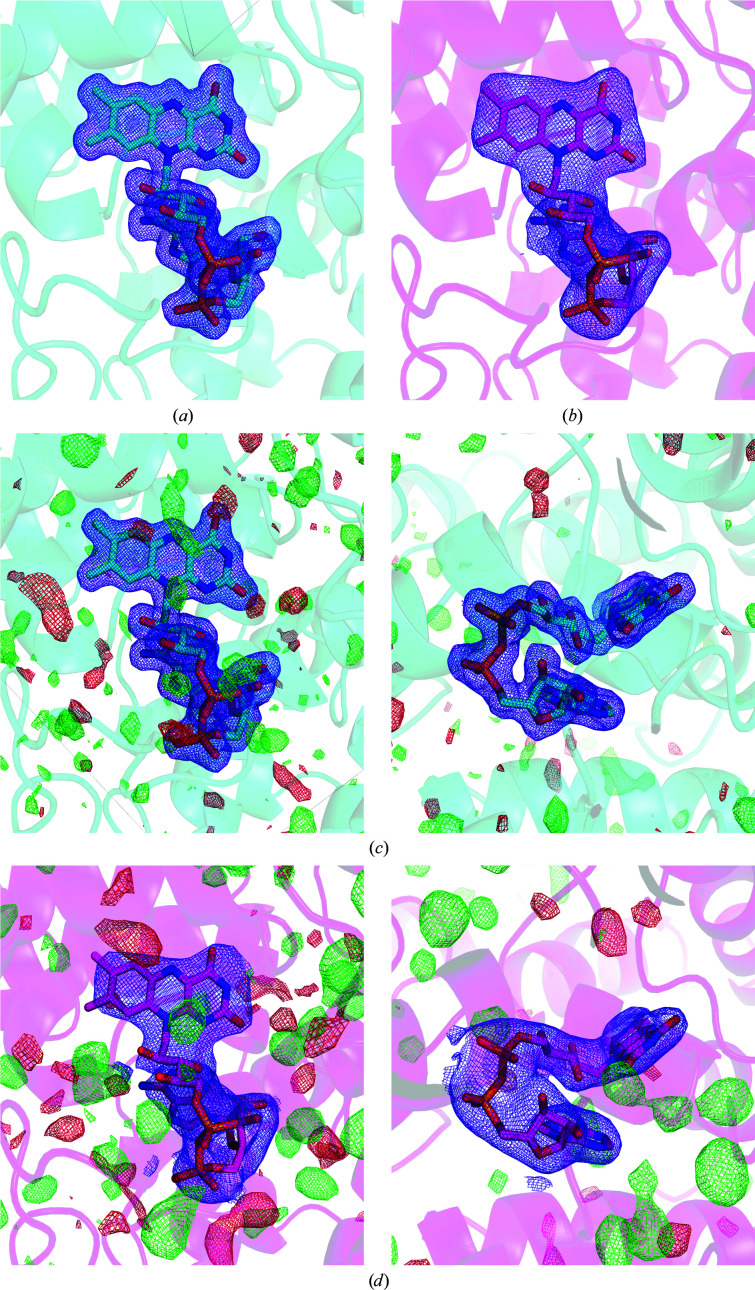
FAD polder maps from the cryo-temperature (*a*) and room-temperature (*b*) structures. The maps are contoured at 3σ. The FAD isoallozaxine ring is planar in both structures. In the lower panel, 1σ 2*F*_o_ − *F*_c_ (blue) and 3σ *F*_o_ − *F*_c_ maps (negative features are in red and positive features are in green) for the cryotemperature (*c*) and room-temperature (*d*) structures are displayed.

As in previous structures of photolyases and cryptochromes (Maul *et al.*, 2008 ▸; Zoltowski *et al.*, 2011 ▸; Park *et al.*, 1995 ▸), our structures at room and cryogenic temperatures show a FAD chromophore with 100% occupancy (Fig. 3 ▸). Several conserved interactions between protein residues and the isoalloxazine ring, ribityl chain, pyrophosphate and adenosine moieties of FAD are observed. Various published X-ray crystallographic structures of photolyases showed that FAD was reduced by X-rays during data acquisition and it should be confirmed whether this is the case (Kort *et al.*, 2004 ▸; Mees *et al.*, 2004 ▸). The X-ray pulses at free-electron lasers are so short that diffraction is recorded before absorbed X-rays can do damage to the protein structure (Neutze *et al.*, 2000 ▸), and we therefore presume that our SFX structure is not subject to radiation damage. Moreover, the bending of its isoalloxazine moiety has been used as an indicator of FAD reduction (Røhr *et al.*, 2010 ▸). In our SFX structure the isoalloxazine atoms occupy the same plane, and butterfly bending along the N5–N10 plane was not observed. Even the cryo-structure shows an unbent FAD. We conclude that the FAD is in a fully oxidized state in both of our structures (Fig. 3 ▸).

In a previous study, the reduction of FAD by X-rays was reported (Kort *et al.*, 2004 ▸). We therefore estimated the average diffraction-weighted X-ray dose during data acquisition to be approximately 3.2 MGy, which is a moderate dose in crystallography. In the previous experiment the crystals were subjected to approximately 2 × 10^15^ photons mm^−2^, but the diffraction-weighted X-ray dose was not reported (Kort *et al.*, 2004 ▸). It is therefore difficult to directly compare the experiments and it remains unclear why this difference in observations arises.

**Figure 4:**
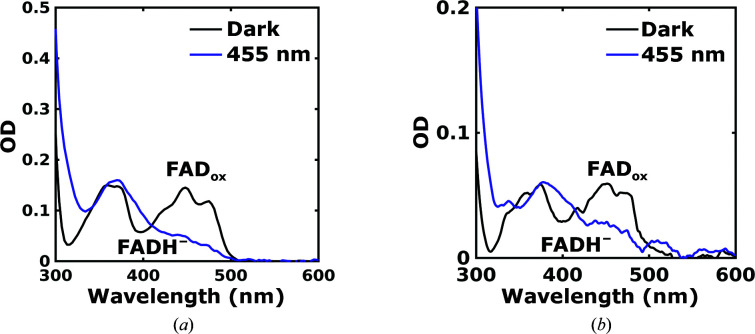
UV–Vis spectrum of *Dm*(6–4)PL. (*a*) UV–Vis spectrum of *Dm*(6–4)PL in solution before (black plot) and after (blue plot) illumination with a 455 nm LED light. (*b*) UV–Vis spectrum of *Dm*(6–4)PL in crystalline form before (black plot) and during (blue plot) illumination with a 455 nm LED light. The measurements were performed at room temperature.

Before performing a time-resolved crystallography experiment, it is important to determine whether the protein is functional in the crystals. We recorded UV–Vis spectra of the *Dm*(6–4)PL protein in solution and microcrystals in order to assess whether the proteins are light-active (Fig. 4 ▸). In the resting state the spectrum shows absorption bands between 400 and 500 nm both in solution and in the crystals. The shape with three peaks is characteristic of FAD in the fully oxidized state (Payne *et al.*, 1990 ▸; Jorns *et al.*, 1990 ▸). Upon photo-illumination with light at 455 nm wavelength, these bands bleach and new absorption bands form at 350–400 nm. This spectral shape is characteristic of fully reduced FADH^−^ (Kao *et al.*, 2008 ▸; Schwinn *et al.*, 2020 ▸). This reduction requires that two photons are absorbed and we therefore infer that the semiquinone ( 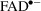 ) is formed as an intermediate. The spectra of the microcrystal slurry were noisier compared with the solution due to significant scattering (Fig. 4 ▸ *b*). However, after subtraction of the scattering background they resembled the shape of those from protein in solution. We therefore conclude that the protein is fully photoactive in solution and in the crystals.

## 4. Discussion   

Several cryo-structures of *Dm*(6–4)PL have been reported (PDB entries 2wb2, 2wq6, 2wq7, 3cvy, 3cvu, 3cvv and 3cvx; Maul *et al.*, 2008 ▸; Glas *et al.*, 2010 ▸). The cryo-structure reported in this paper increases the resolution to 1.79 Å. This is the highest resolution reported for *Dm*(6–4)PL in the PDB.

At cryogenic temperature, many biologically relevant reactions can be hindered. For example, the electron-transfer events in wild-type photosynthetic reaction center from *Blastochloris viridis* have been observed to be drastically inhibited below 215 K (Ortega *et al.*, 1998 ▸). Also, the DNA-repair activity of photolyase using blue light is suppressed at temperatures below 200 K (Langenbacher *et al.*, 1997 ▸). The conformation of a CPD photolyase complexed with DNA has been captured after *in situ* repair by X-ray-induced reduction during data collection (Mees *et al.*, 2004 ▸). However, crucial intermediates in the photoactivation and DNA-repair process of photolyases upon blue-light activation are still missing. Here, we open up the possibility of recording crystallographic snapshots of *Dm*(6–4)PL at room temperature using time-resolved SFX.

The microcrystals are easy to produce in batch mode and have a homogeneous size distribution of 20–40 µm. The small size and homogeneous size distribution of the crystals allow more uniform illumination of the crystals. Despite their small size, our microcrystals diffracted beyond 2.27 Å, a resolution that is high enough to be able to observe conformational changes of side chains upon blue-light activation and is suitable for a time-resolved pump–probe serial crystallographic experiment. For some X-ray laser facilities it may be necessary to grow even smaller crystals, but we foresee that this may be achieved by further tuning the crystallization conditions.

*Dm*(6–4)PL proteins in microcrystals are in the fully oxidized state as a resting state and remain photoswitchable in the crystal lattice. This indicates that the photoreactions are not hindered by the crystal packing in the microcrystals. From the fully oxidized state to the fully reduced state upon blue-light activation, FAD undergoes reduction twice in *Dm*(6–4)PL. It should be possible to study the single reduction of FAD to semiquinone FAD in *Dm*(6–4)PL, a process that is shared with cryptochromes, using time-resolved serial crystallography (Pande *et al.*, 2016 ▸; Tenboer *et al.*, 2014 ▸). Depending on the time resolution, it may also be possible to follow the electron transfer from light-excited FAD along the four tryptophan residues.

## 5. Conclusion   

Here, we show that *Dm*(6–4)PL microcrystals suitable for SFX studies diffract to high resolution in order to observe conformational changes upon light activation. The availability of well diffracting microcrystals opens up a route to conducting time-resolved serial crystallography experiments on *Dm*(6–4)PL, a member of the photolyase/cryptochrome family, at room temperature. In pump–probe time-resolved experiments, crystal size is crucial for obtaining high-quality data with a good signal-to-noise ratio from light-illuminated states. The reduced crystal size facilitates light dispersion through the crystals and improves the portion of activated proteins. Today, X-ray free-electron laser (XFEL) facilities and synchrotons are equipped for time-resolved crystallo­graphy experiments with various ranges of time delay from femtoseconds to milliseconds. The *Dm*(6–4)PL microcrystals can be employed in these setups to capture the structural conformational changes that occur during photoactivation and DNA repair (Schmidt, 2015 ▸; Weinert *et al.*, 2019 ▸). This may provide opportunities to obtain a much more detailed understanding of the protein response during these crucial processes for life. 

## Supplementary Material

PDB reference: *Dm*(6–4)photolyase, cryogenic temperature, 7ayv

PDB reference: room temperature, 7azt

## Figures and Tables

**Table 1:** Data-collection and refinement statistics Values in parentheses are for the outer shell.

	Cryogenic temperature	Room temperature
PDB code	7ayv	7azt
Data-collection statistics		
Space group	*P*2_1_2_1_2_1_	*P*2_1_2_1_2_1_
*a*, *b*, *c* (Å)	60.90, 88.93, 114.02	61.63, 91.64, 115.97
α, β, γ (°)	90.0, 90.0, 90.0	90.0, 90.0, 90.0
Resolution (Å)	29.42–1.79 (1.85–1.79)	42.61–2.27 (2.35–2.27)
Data completeness (%)	99.2 (89.4)	100.0 (100.0)
*R*_merge_	0.106 (0.831)	N.A.
*R*_split_ (%)	N.A.	12.06 (147.58)
CC_1/2 _	99.9 (83.5)	97.22 (31.64)
〈*I*/σ(*I*)〉	16.90 (2.50)	4.90 (0.78)
No. of reflections	58656 (3836)	33953 (1352)
Multiplicity	12.7 (11.6)	106.4 (21.3)
No. of hits	N.A.	12027
No. of indexed hits	N.A.	10310
Refinement		
*R*_work_/*R*_free_	0.176/0.206	0.172/0.208
Wilson *B* factor (Å^2^)	24.2	90.8
Total No. of atoms	4459	4168
Average *B*, all atoms (Å^2^)	27.0	93.0
R.m.s. deviations		
Bond lengths (Å)	0.015	0.007
Bond angles (°)	1.21	0.91
